# Safety Evaluation of Nano-Liposomal Formulation of Amphotericin B (Sina Ampholeish) in Animal Model as a Candidate for Treatment of Cutaneous Leishmaniasis

**Published:** 2018-09-30

**Authors:** Seyed Ebrahim Eskandari, Alireza Firooz, Mansour Nassiri-Kashani, Mahmoud Reza Jaafari, Amir Javadi, Akram Miramin-Mohammadi, Hossein Valian-Keshavarz, Ali Khamesipour

**Affiliations:** 1 Center for Research and Training in Skin Diseases and Leprosy (CRTSDL), Tehran University of Medical Sciences, Tehran, Iran; 2 Nanotechnology Research Center, Pharmaceutical Technology Institute, Mashhad University of Medical Sciences, Mashhad, Iran; 3 Department of Pharmaceutical Nanotechnology, School of Pharmacy, Mashhad University of Medical Sciences, Mashhad, Iran; 4 School of Allied Sciences, Tehran University of Medical Sciences, Tehran, Iran; 5 Department of Medical Parasitology and Mycology, School of Public Health, Tehran University of Medical Sciences, Tehran, Iran; 6 Center for Research of Endemic Parasites of Iran, Tehran University of Medical Sciences, Tehran, Iran

**Keywords:** Cutaneous leishmaniasis, Nano-liposomes, Amphotericin B, Draize test

## Abstract

**Background:**

Development of a topical treatment for cutaneous leishmaniasis (CL) is an important step in the improvement of lesion management. Amphotericin B (AmB) is effective against *Leishmania* species but it is toxic, a Nano-liposomal form of AmB with a size of about 100nm (Lip-AmB) was developed and showed to be effective against *Leishmania major*, and *Leishmania tropica in vitro* and against *L. major in vivo* in animal model. This study was designed to check the irritancy Draize test in rabbits and was completed in the Center for Research and Training in Skin Diseases and Leprosy, TUMS, in 2012.

**Methods:**

Twenty rabbits in 3 steps were housed individually with artificial lighting (12/12h light/dark). SinaAmpholeish cream or empty liposomes (prepared under GMP condition at Minoo Company, Tehran, Iran), was applied on a gauze patch and the patches were placed on the designated sites of the skin in the back of the rabbits. At 48 and 72h later, the erythema and oedema were checked, scored and recorded.

**Results:**

The erythema score in rabbits was 0.83+0.41 for the SinaAmpholeish and 0.5+0.55 for empty liposomes (P= 0.16). The average score for oedema was 0.67+0.52 for SinaAmpholeish and 0.33+0.52 for empty liposomes (P= 0.16).

**Conclusion:**

Based on skin irritancy reactions the topical formulation of SinaAmpholeish is safe and could be further checked in human trials.

## Introduction

Cutaneous leishmaniasis (CL) is endemic in 88 countries (72 of them are developing countries and 16 developed ones) and 90% of CL cases are reported from 6 countries including Brazil, Peru, Afghanistan, Saudi Arabia, Syria and Iran (1–3). Pentavalent antimonial derivatives (sodium stibogluconate=Pentostam and meglumine antimoniate=Glucantime) are still considered as the standard first-line treatment for CL. Currently available treatments are far from acceptable criteria either by the patients or by the health authorities due to pain at the site of injections, high risk of side effects, high cost and low efficacy, moreover resistant is reported from endemic areas. The development of new treatment is highly demanded, invention of a topical treatment for CL is very essential to increase the compliance rate and control of anthroponotic CL (4–7).

Amphotericin B (AmB) is a polyene antibiotic produced from *Streptomyces nodosus*. Several formulations of AmB have been marketed and used to treat fungal infections and visceral leishmaniasis, but the main drawback is toxicity. AmBisome™ is a liposomal form of AmB which is the first line treatment for visceral leishmaniasis in some endemic areas but is not as effective against CL (8–11). A nano-liposomal form of amphotericin B (Lip-AmB) with a size of about 100nm was formulated and the method of preparation is under US Patent. The formulation is effective against *Leishmania major* and *Leishmania tropica in vitro* and against *L. major in vivo* in a murine model of leishmaniasis (9). Large-scale production of 0.4% Lip-AmB was done under Good Manufacturing Practice guidelines under the trade name of SinaAmpholeish at Minoo Company of (Tehran, Iran).

Draize test was initially introduced to test various medical, cosmetic, and chemical substances, but was also later used to check the safety of several other products such as cream, lipstick, mascara, face lotion, soap, shampoo, powder, hairspray, toothpaste, lacquer, detergent and anything, which could possibly induce skin irritation (10). It has been modified and updated several times and the most commonly used method is the one introduced by Federal Hazardous Substance Act, Title 16, Chapter II, reference Consumer Product Safety Commission 1980 as a standard test to check skin irritancy reactions. The rabbit is the most commonly used animal for skin irritation test and usually, 6 rabbits are used (11–14).

The in vivo rabbit eye irritation/corrosion test developed from Draize skin test since 1981. It is used for two types of substances that either might be exposed to the eye due to regular use in or around the eyes (such as eye creams) or through accidental exposure of the eyes to the products that are not originally designed to be in contact with eyes (such as shampoos or skin creams). The evaluation of eye irritation potential for cosmetic substances is necessary to assure that the products are safe for consumers if eyes are exposed to intentionally or accidentally. Usually, 0.1 ml (or weight equivalent approximately 100mg) of the test substance is placed into one eye and the other eye is not treated and kept as control. The treated eye and the control eye are checked regularly after application of the product, usually 1, 24, 48 and 72h after exposure or more frequently if required. Eye irritation is defined as induction of pathological changes in the eye fully recovered within 21d or tissue damages in the eye which might not fully recover even within 21d (14, 15).

The objective of this study was to evaluate the safety and irritancy potential of SinaAmpholeish in rabbit model (Draize test) as well as an eye irritation test, as a step prior to clinical trials in humans.

## Materials and Methods

The study was conducted in 2012 in the Center for Research and Training in Skin Diseases and Leprosy, TUMS, Tehran, Iran. The proposal of this study was approved by the Ethical Committee of Center for Research and Training in Skin Diseases and Leprosy, Tehran University of Medical Sciences, Tehran, Iran

Five-month-old male and female rabbits were purchased from Razi Vaccine and Serum Research Institute, Hesarak, Iran and kept in the animal house of CRTSDL. Rabbits were housed individually with artificial lighting (12h light, 12h dark) and free access to conventional laboratory diets and an unrestricted supply of drinking water with a collar fitted and fastened around the rabbit’s neck.

The irritation test was performed in 3 steps:

**Step 1)** Healthy white rabbits with no hair loss were purchased and hairs on the back of the animals were shaved. After 24h, 6 rabbits with no sign of abrasion, erythema or oedema due to shaving were selected, 500mg of either 0.4% SinaAmpholeish gel or empty liposomes (prepared under GMP condition at Minoo Company, Tehran, Iran), was applied on a gauze patch and the patches were placed on the designated sites of the skin in the back of the rabbits, secured and moistened using 0.5 ml of phosphate buffer saline (PBS). The positive control site was covered with a patch containing 0.5ml of 1% of sodium lauryl sulfate (SLS). The negative control site was covered with a patch containing 0.5ml of PBS. Then the entire back of the rabbits was covered for 24h.
  After 24h of wrapping, the patches were removed. At one hour after removal of the patches and at 48 and 72h later, the erythema and oedema were checked, scored and recorded (Table 1).
**Step 2)** Healthy New Zealand Albino rabbits were screened and rabbits with no hair loss were selected. The hairs on the back of the rabbits were clipped.
  After 24h, 6 rabbits with no signs of abrasion, erythema or oedema due to shaving were selected and the following materials were applied on their back: SinaAmpholeish gel, empty liposome (as placebo), SLS 1% as a positive control and PBS as negative control similar to step 1.
**Step 3)** In a pilot study, first white but not New Zealand Albino rabbits were screened and 2 healthy rabbits with no ocular lesion was selected, after gently pulling the lower lid away from the right eyeball, 100mg of SinaAmpholeish gel was placed in the conjunctival sac, then lids were then gently held together for about one second in order to prevent loss of the material. The left eye was untreated as a control. The eyes of the rabbits were regularly checked for 24h and in 2 of them daily for one week. As there was no sign of pain (such as repeated pawing or rubbing of the eye, excessive blinking, excessive tearing), no anaesthesia was required for the other rabbits.

**Table 1.** Rabbit score values for skin reactions (erythema and oedema)

**Skin reaction**	**Value**
**Erythema formation:**	
No erythema	0
Very slight erythema (barely perceptible)	1
Well-defined erythema	2
Moderate to severe erythema	3
Severe erythema (beet redness) to slight scar formation (injuries in depth)	4
Necrosis (death of tissue)	+N
Scar (sloughing or scar formation)	+E
**Oedema Formation:**	
No oedema	0
Very slight oedema (barely perceptible)	1
Slight oedema (edges of area well-defined by definite raising)	2
Moderate oedema (raised approximately one millimetre)	3
Severe oedema (raised approximately one millimetre and extending beyond the area of exposure)	4
Total possible score for primary irritation	8

Than 6 New Zealand Albino rabbits with no sign of ocular lesions were selected and treated similarly to the pilot study in white rabbits.

In 2 rabbits the right eyes were covered for one hour. The eyes of the rabbits were regularly checked for the first 24h and photos were taken at 24, 48 and 72h.

### Statistical analysis

The Wilcoxon signed-rank test was used for comparing reaction between the two arms of study, P-value of less than 0.05 was considered significant.

## Results

Step one results of the irritancy potential test of the substances were checked in 6 white rabbits (but not New Zealand Albino), the results showed no reaction in any of the rabbits, no erythema, and no oedema.

In step 2, irritancy potential test was checked in New Zealand Albino rabbits, as it is shown in Table 2, the average of erythema scores in 6 rabbits in 3-time points was 0.83 +0.41 for 0.4% SinaAmpholeish gel and 0.5+ 0.55 for empty liposomes (P= 0.16). The average scores for oedema in the same rabbits was 0.67+0.52 for SinaAmpholeish gel and 0.33+0.52 for empty liposomes (P 0.16). The results of erythema and oedema are shown in Tables 2 and 3, respectively (Table 3).

**Table 2.** Skin erythema reactions in New Zealand Albino rabbits (Wilcoxon Signed Ranks test)

**Hours**	**Nano Liposomal Amphotericin-B**		**Placebo Empty Liposome**		**P-value**

	**Mean+SD**	**Median+QR**	**Mean+SD**	**Median+IQR**	
**25**	0.83+0.41	1+0.25	0.5+0.55	0.5+1	0.16
**48**	0.5+0.55	0.5+1	0	0	0.08
**72**	0	0	0	0	1

Sodium Lauryl Sulfate (SLS) was used as a positive control and PBS as a negative control Positive control results were 3, 4, 3 at 25, 48 and 72h and negative control was 0

**Table 3.** Skin oedema reactions in New Zealand Albino rabbits (Wilcoxon Signed Ranks test)

**Hours**	**Nano Liposomal Amphotericin-B**		**Placebo Empty Liposome**		**P-value**

	**Mean+SD**	**Median+IQR**	**Mean+SD**	**Median+IQR**	
**25**	0.67+0.52	0	0.33+0.52		0.16
**48**	0	0	0	0	1
**72**	0	0	0	0	1

Sodium Lauryl Sulfate (SLS) was used as a positive control and PBS as a negative control

**Fig. 1.**
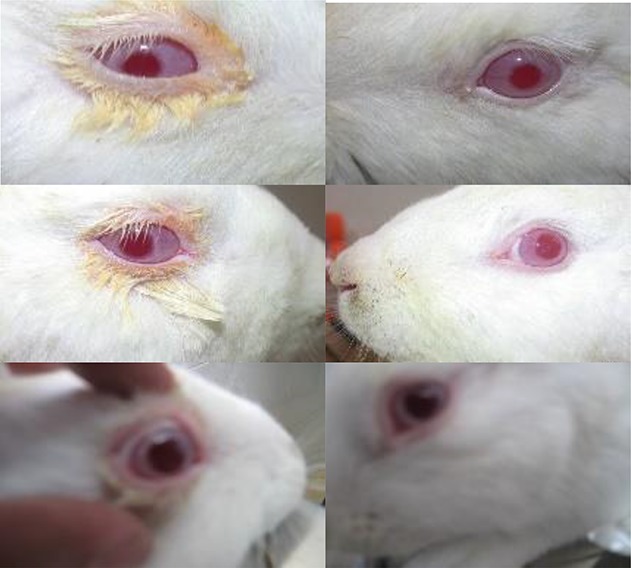
Pictures of eye irritation test, left experiment, right control. Positive control results were 2, 2, 2 at 25, 48 and 72h and negative control showed was 0 at all time frames

No sign of eye irritation was seen during 72h observation in 8 rabbits (two from the pilot study and 6 from the main study) and two-week observation in 2 rabbits (Fig. 1).

## Discussion

Treatment of CL is sometimes a dilemma, the only standard treatment is to use pentavalent antimonite which needs multiple injections, accompanies side effects and moreover is not always effective. Development of new more acceptable treatment for CL is urgently needed to increase compliance and facilitate control of anthroponotic form of CL. Topical formulation is an ideal treatment for CL, several topical ointment have been developed and checked in phase 3 clinical trials (4, 6, 13, 15–17).

Paromomycin had therapeutic effect on old world and new world CL, but currently, none is available in the market for endemic areas (17). Various modalities including even immunotherapy have been proposed to treat CL with limited efficacy (18). A Nano-liposomal form of amphotericin B was developed. This formulation showed to be effective against *L. major* and *L. tropica in vitro* and against *L. major in vivo* in a murine model of leishmaniasis (9).

The risk of topical formulations such as cosmetic and therapeutic to induce irritation or corrosion is usually checked in rabbit using Draize test (12–14). An alternative method has been developed to decrease and even avoid using animal models for experiment studies (13). Originally it was proposed to use alternative *in vitro* test to check the irritancy test but due to politically imposed sanctions against Iran, the cell line needed to use *in vitro* test was not available so with no other option, the formulation of Nano-liposomal Amphotericin B potential irritancy was checked in rabbit model (Draize test).

According to our previous unpublished data, usually New Zealand Albino rabbit is more sensitive to irritancy test than white rabbits, so first 6 white rabbits were included and no erythema and no oedema was seen in 25, 48 and 72h exposure to either SinaAmpholeish or empty liposomes. When the same procedure was used in 6 New Zealand Albino rabbits the average of erythema and oedema scores was 0.83+0.41 and 0.67+0.52, respectively for SinaAmpholeish gel and 0.5+0.55 and 0.33+ 0.52 for erythema and oedema for empty liposomes, respectively, although there was no significant difference between irritancy test between SinaAmpholeish gel and empty liposomes and control groups (P=0.16), but New Zealand Albino rabbits were more sensitive than white rabbits but the formulation was completely safe and could be further checked in human trials.

## Conclusion

Based on skin irritancy reactions the topical formulation of SinaAmpholeish is safe and could be further checked in human trials.
